# The effect of C-terminal deamidation on bacterial susceptibility and resistance to modelin-5

**DOI:** 10.1007/s00249-025-01732-4

**Published:** 2025-02-11

**Authors:** Sarah R. Dennison, Leslie H. G. Morton, Kamal Badiani, Frederick Harris, David A. Phoenix

**Affiliations:** 1 Biomedical Evidence-Based Transdisciplinary (BEST) Health Research Institute, School of Pharmacy and Biomedical Sciences, University of Central Lancashire, Preston, PR1 2HE UK; 2 Biosynth Ltd, 4 Feldspar Close, Warrens Park, Enderby, Leicestershire, LE19 4JS UK; 3 Office of the Vice Chancellor, London South Bank University, 103 Borough Road, London, SE1 0AA UK

**Keywords:** Modelin-5, α-helical structure, C-terminal amide, *Escherichia coli*, *Staphylococcus aureus*, Lys-PG

## Abstract

The C-terminal amide carried by antimicrobial peptides (AMPs) can play a variable role in their antibacterial action and here, this role is investigated here for the synthetic peptide modelin-5 (M5-NH_2_). The peptide showed potent activity against *Pseudomonas aeruginosa* (MLC = 5.9 µM), with strong binding to the cytoplasmic membrane (CM) (K_d_ = 21.5 μM) and the adoption of high levels of amphiphilic α-helical structure (80.1%) which promoted strong CM penetration (9.6 mN m^−1^) and CM lysis (89.0%). In contrast, *Staphylococcus aureus* was resistant to M5-NH_2_ (MLC = 139.6 µM), probably due electrostatic repulsion effects mediated by Lys-PG in the organism’s CM. These effects promoted weak CM binding (K_d_ = 120.6 μM) and the formation of low levels of amphiphilic α-helical structure (30.1%), with low levels of CM penetration (4.8 mN m^−1^) and lysis (36.4%). C-terminal deamidation had a variable influence on the antibacterial activity of M5-NH_2_, and in the case of *S. aureus*, loss of this structural moiety had no apparent effect on activity. The resistance of *S. aureus* to M5-NH_2_ isoforms appeared to be facilitated by the high level of charge carried by these peptides, as well as the density and distribution of this charge. In the case of *P. aeruginosa,* the activity of M5-NH_2_ was greatly reduced by C-terminal deamidation (MLC = 138.6 µM), primarily through decreased CM binding (K_d_ = 118.4 μM) and amphiphilic α-helix formation (39.6%) that led to lower levels of CM penetration (5.1 mN m^−1^) and lysis (39.0%).

**Supplementary Information:**

The online version contains supplementary material available at 10.1007/s00249-025-01732-4.

## Introduction

The view that innate immunity involved direct antimicrobial activity was born in the latter decades of the ninteenth century (Martins et al. 2023); however, it was the twentieth century before a series of landmark studies showed that key effectors of this activity were endogenous antibiotics and named antimicrobial peptides (AMPs) (George et al. 2023; Phoenix et al. 2013a). Major examples of these studies include the isolation of cecropin from moths (Hultmark et al. 1980; Steiner et al. 1981) and magainin from frogs in the 1980s (Zasloff 1987) which was followed in the 1990s by the discovery of human LL-37 (Gudmundsson et al. 1996) and beta-defensins (Bensch et al. 1995; Harder et al. 1997), as well work on fruit flies demonstrating the relevance of AMPs to host defence (Lemaitre et al. 1996). Since these studies, approaching 4000 AMPs have been identified, with representatives produced in virtually all multicellular organisms, as evidenced in the many databases created to list these peptides and analyse their properties (Ramazi et al. 2022; Zhang et al. 2023).

In general, AMPs do not affect the microbiota and healthy cells of the host and primarily contribute to innate defence by eliminating pathogenic microbes, including bacteria, fungi, and viruses, although other defence roles, such as immunomodulatory functions, are increasingly being reported (Huan et al. 2020; Talapko et al. 2022). It is well established that in the course of their antibacterial activity, AMPs must pass through the various envelopes that encompass bacterial cells to reach their primary site of action, which is the cytoplasmic membrane (CM) of these cells (Gan et al. 2021; Phoenix et al. 2013b). In the case of Gram-negative bacteria, AMPs first encounter and then need to traverse the barrier posed by the outer membrane (OM) which is primarily composed of anionic lipopolysaccharide (LPS) (Barreto-Santamaría et al. 2021; Silhavy et al. 2010). Three major routes to achieve this passage are available: diffusion directly through the OM or via porins, or translocation through electrostatic interaction with LPS and self-promoted uptake (Sharma and Ayappa 2022; Ude et al. 2021). In the case of Gram-positive bacteria, AMPs initially encounter the barrier of the cell wall, which is essentially a thick layer of peptidoglycan that is decorated with anionic teichoic acids and lipoteichoic acids (Malanovic and Lohner 2016a, b). In general, AMPs are able to diffuse through the cell wall of Gram-positive bacteria, and the much thinner cell wall of Gram-negative bacteria, which is sandwiched between the OM and CM (Li et al. 2017a; Silhavy et al. 2010). This diffusion can facilitate the accumulation AMPs on the surface of the CM due to favourable interactions between anionic components of cell walls and the positive charges carried by these peptides (Malanovic and Lohner 2016a, b). Having accessed the CM, the positive charge and amphiphilic structures possessed by these peptides, promote interactions with lipid components of this membrane and thereby, a variety of mechanisms that lead to bacterial death (Li et al. 2021; Zhang et al. 2021). These mechanisms drive permeabilization of the CM, for example, the Carpet and Toroidal pore models, and / or translocation to attack intracellular components, as in the case of the SHM model (Kumar et al. 2018; Phoenix et al. 2013b; Zhang et al. 2021). In addition, rather than direct interaction with lipid, some AMPs are known use CM protein transporters to pass through this membrane and enter bacterial cells to act on cellular targets (Graf and Wilson 2019). The generally non-specific nature of these antimicrobial mechanisms allows AMPs to bypass the mechanisms used by bacteria to resist conventional antibiotics and minimises the likelihood of these organisms developing resistance to AMPs (Rima et al. 2021; Xuan et al. 2023). Based on these properties, these peptides have been investigated as highly attractive contenders to treat infections due to bacterial pathogens (Huan et al. 2020; Talapko et al. 2022) in areas ranging from biofilm eradication (Zhu et al. 2022) to topical and systemic application (Wang and Mechesso 2022).

Despite the clear therapeutic potential of AMPs, a number of problems have beset the full achievement of this potential, with the result that although many of these peptides have been clinically trialled, only a small number are in clinical use (Dijksteel et al. 2021; Lesiuk et al. 2022). In response a number of strategies to improve the efficacy of AMPs have been utilised (Kang et al. 2022; Wang et al. 2023), including the design of synthetic peptides based on the sequences of their naturally occurring counterparts (Lima et al. 2021). A sequence feature of naturally occurring AMPs that is often incorporated into the design of their synthetic mimics is the inclusion of residues with post-translational modifications (PTMs) (Cardoso et al. 2021; Gan et al. 2021). These modified residues play a key role in promoting the antimicrobial activity of AMPs and currently, the APD3 database includes peptides that collectively exhibit over twenty PTMs (Gan et al. 2021; Wang et al. 2016), ranging from halogenation to lipidation (Bellavita et al. 2023; Mardirossian et al. 2021). One of the most extreme cases of these AMPs would appear to be styelin D from the solitary tunicate, *Styela clava*, which possesses around twelve individually modified residues that help promote its activity against marine bacteria (Malik et al. 2016). However, amongst the most frequently occurring groups of PTMs reported for AMPs are those involved in end-capping effects, including C-terminal amidation (Gan et al. 2021), which is particularly prevalent in amphibian peptides (Wang 2020; Xu and Lai 2015). These PTMs are also those most frequently incorporated into designed, synthetic AMPs (Gan et al. 2021; Han et al. 2021) and one of the most prominent examples of these peptides is pexiganan, which was based on the sequence of magainin (Dennison et al. 2014b). Characterisation showed pexiganan to possess potent, antibacterial membranolytic action that was enhanced by C-terminal amidation (Gottler and Ramamoorthy 2009), and led to its position as one of the first synthetic α-helical AMPs to be patented (Kang et al. 2017) and enter clinical trials (Dijksteel et al. 2021; Dumville et al. 2017). Pexiganan had limited success in phase III clinical trials for the treatment of diabetic foot ulcers (Greber and Dawgul 2017); however, recent research has shown that switching the peptide bond of cationic residues in pexiganan to amide bond isosteres led to superior antibacterial efficacy and clear therapeutic potential (Wani et al. 2021).

Another synthetic α-helical peptide derived from magainin is modelin-5, which, similarly to pexiganan, is C-terminally amidated (M5-NH_2_) and has been patented (Owen 2005), based on its potent, selective antimicrobial activity (Dennison and Phoenix 2011a, 2011b; Dennison et al. 2019; Owen 2005). However, in contrast to pexiganan (Gottler and Ramamoorthy 2009), mechanisms underpinning the antimicrobial activity of M5-NH_2_ have only been investigated in a few cases and here, the activity of the peptide against *Pseudomonas aeruginosa* is studied (Dennison and Phoenix 2011a; Dennison et al. 2019). In further contrast to pexiganan (Gottler and Ramamoorthy 2009), *Staphylococcus aureus* shows resistance to M5-NH_2_ using unknown mechanisms, which are also investigated here, and in the case of both bacteria, the potential role of the peptide’s C-terminal amide moiety is a major focus of these investigations (Bessalle et al. 1993; Owen 2005).

## Methods and materials

### Materials

M5-NH_2_ and its non-amidated isoform M5-OH were supplied by Pepceuticals (Leicestershire, UK), purified by HPLC to purity greater than 99% and their sequences confirmed by MALDI mass spectrometry, as KLAKKLAKLAKLAKAL-CONH_2_ and KLAKKLAKLAKLAKAL-COOH, which have charges of + 7 and + 6, respectively (Table 1) (Bessalle et al. 1993; Owen 2005). The phospholipids used were POPG 1-palmitoyl-2-oleoyl-sn-glycero-3-phosphoglycerol; TOCL (1,1',2,2'-tetraoleoyl cardiolipin): 1',3'-bis[1,2-dioleoyl-sn-glycero-3-phospho]-glycerol; POPE: 1-palmitoyl-2-oleoyl-sn-glycero-3-phosphoethanolamine; POPC (1-palmitoyl-2-oleoyl-sn-glycero-3-phosphocholine) and Lys-PG (Lys-DOPG): 1,2-dioleoyl-sn-glycero-3-[phospho-rac-(3-lysyl(1-glycerol))], all of which were purchased from Avanti Polar Lipids (Alabaster, AL). M 4-(2-hydroxyethyl)−1-piperazineethanesulfonic acid (HEPES) and ethylenediaminetetraacetic acid (EDTA) were purchased from Merk Sigma-Aldrich. All buffers were prepared using ultra-pure water (resistivity 18 MΩ cm). Ringer’s solution, nutrient broth and nutrient agar were purchased from Thermo Fisher Scientific (Leicestershire, UK). HPLC grade solvents were obtained from VWR International Ltd (Lutterworth, UK), whilst calcimycin (A23187) and all other regents were purchased from Merck Sigma-Aldrich Company Ltd (Dorset, UK).

**Table 1:** Charge-based characteristics of α-helical AMPs ineffective against *S. aureus*

AMPs	Charge	Sequences	Charge density	MLC (µM)
M5-NH_2_	+ 7	KLAKKLAKLAKLAKAL-NH_2_	0.43	139.6
M5-COOH	+ 6	KLAKKLAKLAKLAKAL	0.37	133.3
CP7	+ 7	ILKKITKLISKLTKKLTK	0.38	150.0
AMH	+ 9	KQKLAKLKALQKLKQKLAKL	0.45	150.0

Table 1 shows the sequence, charge and charge density of synthetic α-helical AMPs to which *S. aureus* has resistance. The charge density of these AMPs is defined as the average net charge per residue and ineffectiveness against *S. aureus* is taken as an MLC (minimum lethal concentration) of greater than 100 µM, as described in (Islam et al. 2023; Zouhir et al. 2016). Data for M5-NH_2_ and M5-OH were derived from this study and (Dennison and Phoenix 2011a), and that for CP7 and AMH from (Hammond et al. 2019; Rakowska et al. 2013; Simcock et al. 2020)

### The preparation of bacterial cultures

Cultures of *P. aeruginosa*, strain NCIMB 8295, and *S. aureus*, strain NCIMB 6571, which had been freeze-dried in 20% (v/v) glycerol and then stored at − 80 °C, were used to inoculate 10 ml aliquots of sterile nutrient broth. These samples were then incubated in an orbital shaker (100 rpm; 37 °C) until the exponential phase (OD = 0 0.6; λ = 6 00 nm) was reached. Each bacterial suspension was centrifuged (15,000 × g; 10 min) to form a cell pellet using a bench top centrifuge (ALC PK 120R). The resulting cell pellet was washed three times in ¼ strength Ringer’s solution and then resuspended in 1 ml of a ¼ strength Ringer’s solution to ensure there was starting inoculum density of *circa* 5.8 × 10^8^ CFU / ml^−1^, all as previously described (Dennison et al. 2019).

### The antibacterial activity of peptides

To evaluate the toxicity of M5-NH_2_ and M5-OH to bacterial cells, stock solutions of each peptide in ¼ strength Ringer’s solution (1000 μM) were diluted to give concentrations in the range 3.90 μM to 1000 μM. Aliquots (1000 μl) of either M5-NH_2_ or M5-OH, at each concentration in this range, were then separately inoculated with suspensions of cells from *P. aeruginosa*, strain NCIMB 8295, and *S. aureus*, strain NCIMB 6571, (10 μl), prepared as described above, and incubated overnight (37 °C). As a control, cultures of these bacteria were similarly treated but in the absence of M5-NH_2_ and M5-OH. After incubation, aliquots of control cultures and peptide treated cultures (10 μl) were spread onto the surface of Nutrient Agar plates and incubated overnight (37 °C; 12 h). After incubation, the plates were viewed and samples with the lowest concentration of M5-NH_2_ and M5-OH yielding no bacterial growth were identified as the minimal lethal concentration (MLC). These experiments were repeated four times and the average MLC determined, as previously described (Dennison and Phoenix 2011a).

### The preparation of small unilamellar vesicles

The CM of *P. aeruginosa*, strain NCIMB 8295, and *S. aureus*, strain NCIMB 6571, were represented by small unilamellar vesicles (SUVs) formed from lipid mixtures with the compositions shown in Table 2. Due to its labile nature, SUVs that included Lys-DOPG were prepared and used immediately (Wölk et al. 2020). These lipid mixtures and either TOCL, POPG or POPE were separately dissolved in chloroform (1 mg ml^−1^), dried under N_2_ gas and then vacuum-dried (4 h), after which the resulting lipid films were rehydrated using 1 × phosphate buffered saline (PBS; pH 7.5). These rehydrated samples were then vortexed (5 min) and the resulting lipid suspensions sonicated (30 min) using a sonicator (Soniprep 150, ISTCP, USA) until clear, followed by three cycles of freeze / thawing. The resulting solutions of SUVs were then extruded eleven times through a 0.1 µm polycarbonate filter using a polar lipids mini-extruder apparatus (Avanti, UK) and diluted ten-fold using 1 × PBS (pH 7.5).

**Table 2:** Lipid composition of bacterial membranes

Bacteria	CM lipids			
	CL (mol %)	POPG (mol %)	POPE (mol %)	Lys-PG (mol %)
*P. aeruginosa*	21	11	68	0
*S. aureus*	5	57	0	38

Table 2 shows the lipid compositions of monolayers and SUVs used to represent the CM of *S. aureus* and *P. aeruginosa*, which were adapted from (Malanovic and Lohner 2016b)

### The conformational behaviour of peptides

Circular dichroism analysis of either M5-NH_2_ or M5-OH was investigated using a J-815 spectropolarimeter (Jasco, UK) at a temperature of 20 °C, which was maintained using a Peltier controller. SUVs formed from either TOCL, POPG or POPE, and those mimicking the CM of *P. aeruginosa*, strain NCIMB 8295, and *S. aureus*, strain NCIMB 6571, were prepared as described above. Due to its labile nature, SUVs that included Lys-DOPG were prepared and used immediately (Wölk et al. 2020). Spectral measurements were performed in 10 mm path length quartz cells (Starna Scientific, UK), which contained these SUVs mixed with stock M5-NH_2_ solution (final concentration of 0.1 mg ml^−1^ in PBS (10 mM; pH 7.4) to give samples with a peptide: lipid molar ratio of 1: 20. Both in the presence and absence of these SUVs, far-UV CD spectra were collected for M5-NH_2_ and M5-OH, where ten scans per sample were obtained using a 10-mm path-length cell. Each scan was performed over a wavelength range of 180 nm to 260 nm at 0.5-nm intervals employing a bandwidth of 1 nm and at a speed 10-nm min^−1^, all as previously (Dennison and Phoenix 2011a). For all spectra obtained, the baseline acquired in the absence of peptide was subtracted and the percentage α-helical content of M5-NH_2_ and M5-OH estimated using the CDSSTR method (protein reference set 3) from the DichroWeb server (Miles et al. 2022). These experiments were repeated four times and the percentage α-helicity of peptides averaged, as previously described (Dennison et al. 2015).

### The membrane binding of peptides

The ability of M5-NH_2_ and M5-OH to bind to bacterial membranes was investigated using a fluorescent probe assay, as previously described essentially, fluorescence was recorded using an FP-6500 spectrofluorometer (Jasco, UK), with an excitation wavelength of 492 nm, an emission wavelength of 516 nm, and excitation and emission slits set to 5 nm. Lipid mixtures with the compositions shown in Table 2 were prepared to mimic the CM of *P. aeruginosa*, strain NCIMB 8295, and *S. aureus*, strain NCIMB 6571. Due to its labile nature, lipid mixtures that included Lys-DOPG were prepared and used immediately (Wölk et al. 2020). These lipid mixtures and either TOCL, POPG or POPE were separately dissolved in chloroform (1 mg ml^−1^) and fluorescein-phosphatidylethanolamine (FPE; 0.5 mol %) added to these solutions, which were then dried under vacuum, overnight, to create lipid films. To form FPE-labelled SUVs, these films were then hydrated with Tris–HCl (10.0 mM; pH 7.4) and EDTA (1.0 mM), followed by freeze–thawing five times and extrusion 11 times through an Avanti mini-extruder apparatus containing a 0.1-µm polycarbonate filter. The efficient incorporation of FPE label into SUVs was confirmed using CaCl_2_ and the calcium ionophore, calcimycin (A23187) (Wall et al. 1995a). To investigate the binding of M5-NH_2_ and M5-OH to the CM of *P. aeruginosa* and *S. aureus*, these peptides (0 to 325 µM) were added to FPE-labelled SUVs mimetic of these CMs, and the fluorescence monitored. The change in fluorescence (ΔF) was then determined as the fluorescence of FPE-labelled SUVs in the presence of peptides minus that of FPE-labelled SUVs in the absence of peptides. These ΔF values were plotted against the concentration of M5-NH_2_ and M5-OH_**,**_ and then fitted by non-linear least squares analysis to Eq. 1:

1 $$\Delta F=(\Delta {F}_{Max }\left[A\right])/({K}_{d}+\left[A\right])$$

where [A] is the concentration of either M5-NH_2_ or M5-OH, ΔF is the change in fluorescence, ΔF_Max_ is the maximum change in fluorescence and K_d_, is the binding coefficient of the peptide (O'Toole et al. 2000; Wall et al. 1995b) obtained by non-linear least squares regression analysis (Maman 2022). These experiments were repeated four times and the average K_d_ for peptides derived, all as previously described (Dennison et al. 2012).

### The membrane partitioning of peptides

The insertion of either M5-NH_2_ or M5-OH into lipid monolayers was investigated at a temperature of 20 °C using a 601 M Langmuir Teflon trough (Biolin Scientific/KSV NIMA, Coventry, UK) equipped with moveable barriers. Lipid mixtures with the compositions shown in Table 2 were prepared to mimic the CM of *P. aeruginosa*, strain NCIMB 8295, and *S. aureus*, strain NCIMB 6571. Lipid mimics of the latter CM were also prepared, except that CL and POPG were replaced by POPC. Due to its labile nature, lipid mixtures that included Lys-DOPG were prepared and used immediately (Wölk et al. 2020). These various lipid mixtures and either TOCL, POPG or POPE were separately dissolved in chloroform (1 mg ml^−1^) and spread dropwise onto the buffer subphase (1 × PBS; pH 7.4) of the 601 M Langmuir trough using a Hamilton syringe and the solvent allowed to evaporate for 10 min. Monolayers were then compressed by the two moveable Derlin barriers of the Langmuir trough at a velocity of 50 mm min^−1^ until a starting surface pressure of 30 mN m^−1^ had been achieved. This surface pressure corresponds to that of naturally occurring cell membranes and is generally used when constructing monolayers representative of bacterial membranes (Dennison et al. 2010, 2014a). Either M5-NH_2_ or M5-OH was injected underneath monolayers to give a final peptide concentration of 6 µM in the subphase, which was maintained at a constant surface area via a built-in controlled feedback system. Changes in surface pressure increases were monitored by the Wilhelmy method using a Whatman’s CH1 filter paper plate and microbalance. These experiments were repeated four times and the changes in maximal surface pressure induced by peptides averaged, as previously described (Dennison et al. 2023).

### Thermodynamic analysis of peptides and their lipid monolayer interactions

Compression isotherms were generated from monolayer mimics of the CM of *P. aeruginosa*, strain NCIMB 8295, and *S. aureus*, strain NCIMB 6571. Chloroformic solutions of lipid mixtures with the compositions shown in Table 2 were prepared (2.5 × 10^15^ molecules) and due to its labile nature, lipid mixtures that included Lys-DOPG were prepared and used immediately (Wölk et al. 2020). These lipid mixtures were spread onto a 1 × PBS buffer subphase (pH 7.4) and the solvent allowed to evaporate for 10 min. Monolayers were left to stabilize for a further 20 min and then the trough barriers were then closed at a speed of 0.22 nm^2^ min^−1^ until monolayer collapse pressure was achieved. Surface pressure changes were monitored and plotted as a function of the area per lipid molecule. Corresponding experiments were then performed except that M5-NH_2_ and M5-OH were separately introduced into the subphase to give a final peptide concentration of 6.0 µM. All experiments were carried out at 20 °C, repeated four times and averaged, as previously described (Dennison et al. 2016).

Thermodynamic analysis of these isotherms was undertaken and used to determine the Gibbs free energy of mixing (ΔG_*mix*_) of monolayers, which provides a measure of the relative stability associated with the miscibility energetics of their pure lipid components. Thermodynamically stable and thermodynamically unstable monolayers are indicated by negative and positive values of ΔG_*mix,*_ respectively (Dennison et al. 2010, 2014a). ΔG_*mix*_ was computed according to Eq. 2:

2 $$\Delta {G}_{mix} = \int [{A}_{\text{1,2}}-\left({X}_{1}{A}_{1}+{X}_{2}{A}_{2}\right)]d\pi$$

where A_1,2_, is the molecular area occupied by the mixed monolayer, A_1_, A_2_ are the area per lipid molecule in the pure monolayers of component 1 and 2, X_1_, X_2_ are the molar fractions of the components and π is the surface pressure. Numerical data were calculated from the compression isotherms according to the mathematical method of Simpson (Todd 1963).

### The membranolytic activity of peptides

The membranolytic ability of M5-NH_2_ and M5-OH was investigated by observing calcein release from SUVs induced by these peptides. Lipid mixtures with the compositions shown in Table 2 were prepared to mimic the CM of *P. aeruginosa*, strain NCIMB 8295, and *S. aureus*, strain NCIMB 6571. These lipid mixtures and either TOCL, POPG or POPE were separately dissolved in chloroform (7.5 mg ml^−1^) and dried in a glass tube to remove the solvent, first under nitrogen and then under vacuum (*circa* 12 h). The resulting dry lipid films were then suspended in HEPES (5.0 mM; pH 7.5) containing calcein (70 mM) and vortexed (5 min) before being sonicated (30 min) using a sonicator (Soniprep 150, ISTCP, USA). To maximise calcein encapsulation, the resulting solutions then underwent five cycles of freeze–thawing before being extruded eleven times through a 0.1-µm polycarbonate filter using a polar lipids mini-extruder apparatus (Avanti, UK). Calcein entrapped in SUVs was then separated from the free dye by elution with HEPES (5.0 mM; pH 7.5) down a Sephadex G75 column (SIGMA, UK), which had been rehydrated overnight in HEPES (20.0 mM; pH 7.5), NaCl (150 mM) and EDTA (1.0 mM).

The calcein release assay was performed by combining 25 ml of SUVs containing entrapped calcein with 50 ml of either M5-NH_2_ or M5-OH (10 mM), which was then made up to a final volume of 1 ml with HEPES (20.0 mM; pH 7.5), NaCl (150 mM) and EDTA (1.0 mM). The fluorescence intensities (FI) of calcein were monitored at 20 °C using an FP-6500 spectrofluorometer (JASCO, UK), with an excitation wavelength of 490 nm and emission wavelength of 520 nm. The fluorescence intensity induced in SUVs containing entrapped calcein by HEPES (20.0 mM; pH 7.5), NaCl (150 mM) and EDTA (1.0 mM) was taken as background leakage and that resulting from the addition of 1 µl of Triton X-100 (10%, v/v) was taken to represent 100% dye release. The percentage lysis induced by M5-NH_2_ and M5-OH was then calculated according to Eq. 3:

3 $$Lysis (\%) \,= \,([FI_{M5-NH2/OH}] - [FI_{HEPES}]) / ([FI_{Triton X}] - [FI_{HEPES}]) \times 100$$

In Eq. 3, the fluorescence of calcein release by peptide is denoted by [FI _M5-NH2/OH_], that released by buffer as [FI _HEPES_] and that released by Triton X-100 as [FI_Triton X_]. These experiments were repeated four times and the percentage lysis achieved by peptides averaged, as previously described (Dennison et al. 2019).

## Results

### The action of M5-NH_2_ and its C-terminally deaminated isoform against*** P. aeruginosa***

Mechanisms underpinning the activity of M5-NH_2_ against *P. aeruginosa* were investigated, and to determine any potential contribution from the peptide’s C-terminal amide moiety, this activity was compared to that of its non-amidated isoform, M5-OH. Consistent with previous studies on the peptide (Owen 2005), M5-NH_2_ showed an MLC of 5.9 µM (Table 3A) against *P. aeruginosa*, which clearly indicated a potent ability to kill the organism that is around ten-fold stronger than that of pexiganan (Gottler and Ramamoorthy 2009). In contrast, M5-OH showed an MLC of 138.6. µM (Table 3A) against *P. aeruginosa*, representing greater than a 20-fold lower ability to kill the organism. In combination, these clearly suggested that the C-terminal amide moiety carried by M5-NH_2_ played a functional role in promoting the peptide’s activity against *P. aeruginosa.* To elucidate the mechanisms involved in this role, major stages in the ability of M5-NH_2_ and M5-OH to interact with the CM of *P. aeruginosa* were studied.

**Table 3:** Properties of M5-NH_2_ and M5-OH interactions with membranes

A. Bacterial CMs				
Membrane	MLC (µM)		K_d_ (µM)	
	M5-NH_2_	M5-OH	M5-NH_2_	M5-OH
*P. aeruginosa*	5.9	138.6	21.5 ± 1.2	118.4 ± 8.3
*S. aureus*	139.6	133.3	120.6 ± 9.0	115.8 ± 10.5

Membrane	α-helicity (%)		Δ*G*_*mix*_	
	M5-NH_2_	M5-OH	M5-NH_2_	M5-OH
*P. aeruginosa*	80.1 ± 0.2	39.6 ± 0.3	> 0	< 0
*S. aureus*	30.1 ± 0.4	29.8 ± 0.5	** < **0	< 0

Membrane	π (mN m^−1^)		Lysis (%)	
	M5-NH_2_	M5-OH	M5-NH_2_	M5-OH
*P. aeruginosa*	9.6 ± 0.8	5.1 ± 0.4	89.0 ± 2.4	39.0 ± 2.8
*S. aureus*	4.8 ± 0.4	4.1 ± 0.2	36.4 ± 2.8	33.7 ± 1.2

B. Individual bacterial lipids				
Membrane	K_d_ (µM)		α-helicity (%)	
	M5-NH_2_	M5-OH	M5-NH_2_	M5-OH
CL	3.7 ± 0.2	10.4 ± 0.4	96.4 ± 0.04	71.0 ± 0.6
PG	6.7 ± 0.2	13.9 ± 0.6	86.1 ± 0.1	61.9 ± 0.1
PE	14.9 ± 0.2	26.9 ± 0.2	53.1 ± 0.1	31.1 ± 0.7

Membrane	π (mN m^−1^)		Lysis (%)	
	M5-NH_2_	M5-OH	M5-NH_2_	M5-OH
CL	12.3 ± 0.4	7.9 ± 0.2	93.1 ± 1.6	60.1 ± 2.4
PG	9.6 ± 0.5	6.1 ± 0.3	74.5 ± 2.7	47.5 ± 1.6
PE	4.9 ± 0.4	3.3 ± 0.2	51.0 ± 2.2	32.2 ± 3.1

**Fig. 1:**
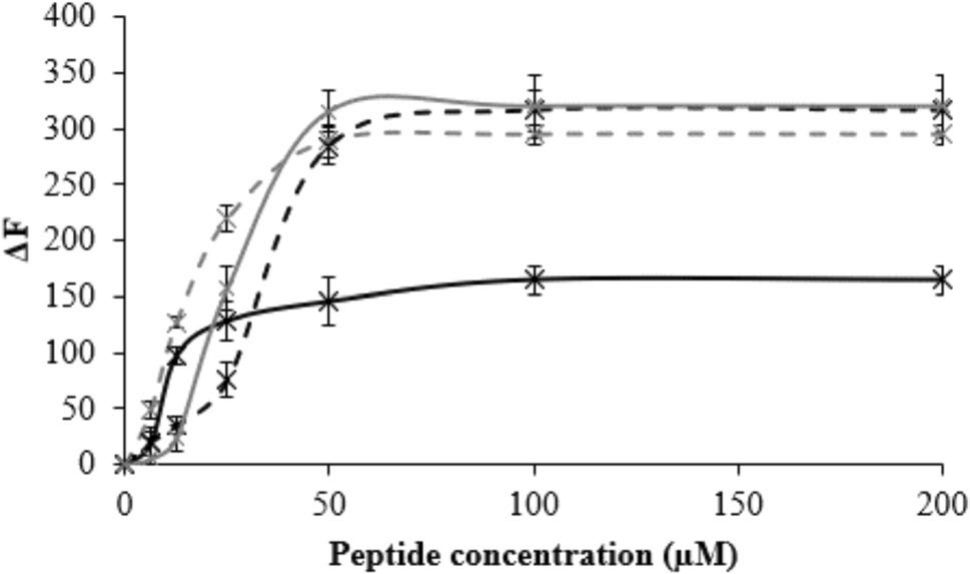
The binding of M5-NH_2_ isoforms to bacterial CMs. Figure 1 shows the change in fluorescence (ΔF) induced by increasing concentrations of M5-NH_2_ with FPE-labelled lipid SUVs that represented the *P. aeruginosa* CM (black line) and the *S. aureus* CM (dashed black line) (Table 2). Also shown are corresponding changes in fluorescence induced by M5-OH with FPE-labelled lipid SUVs representing the *P. aeruginosa* CM (grey line) and the *S. aureus* CM (dashed grey line). In each case, analysis of these curves was used to derive K_d_ (Table 3A), as described above, and error bars represent the standard deviation (Maman 2022)

**Fig. 2:**
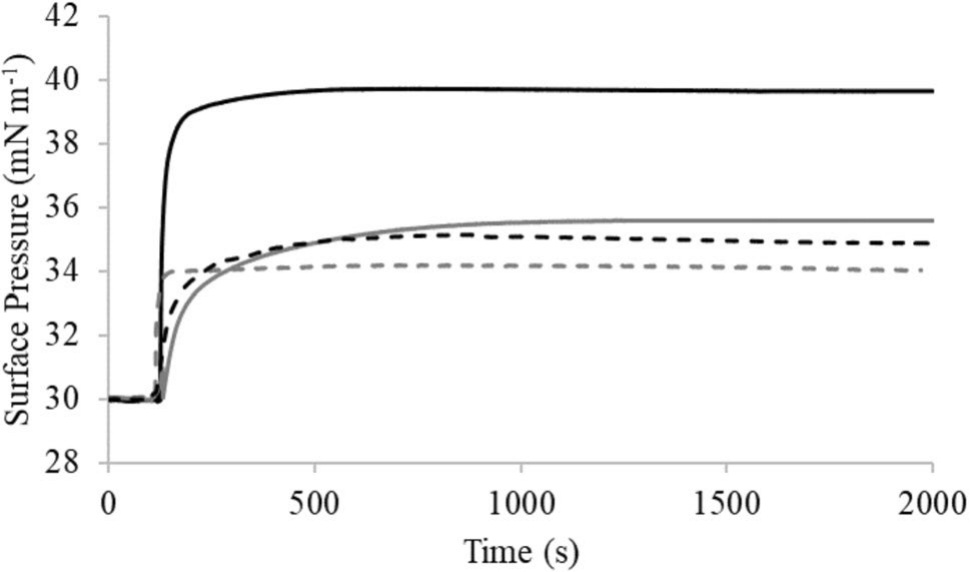
The interaction of M5-NH_2_ isoforms with bacterial CMs. Figure 2 shows the change in surface pressure induced by increasing concentrations of M5-NH_2_ in lipid monolayers that represented the *P. aeruginosa* CM (black line) and the *S. aureus* CM (dashed black line) (Table 3). Also shown are corresponding changes in surface pressure induced by M5-OH with monolayers representing the *P. aeruginosa* CM (grey line) and the *S. aureus* CM (dashed grey line). In each case, maximal surface pressures were determined (Table 3A)

**Fig. 3:**
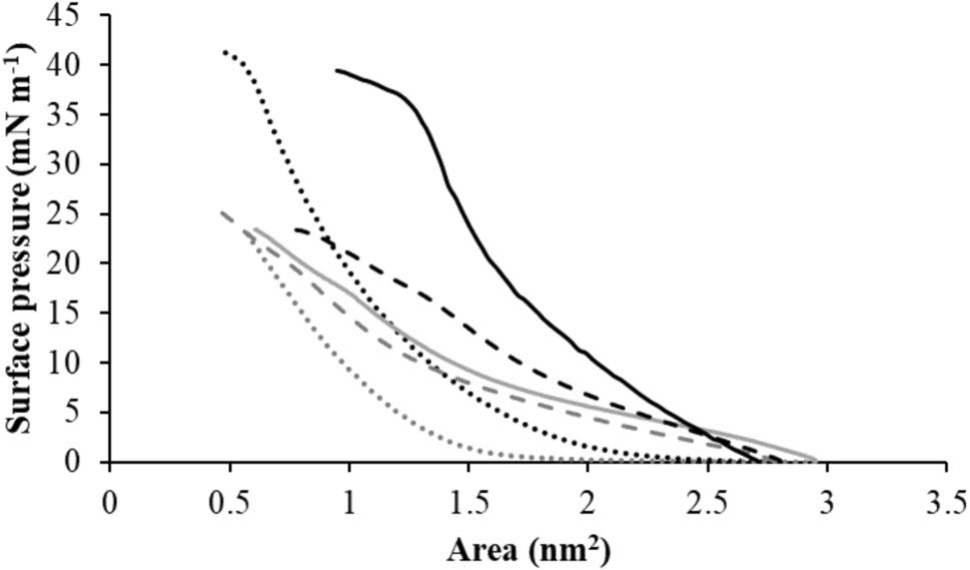
Compression isoform analysis of M5-NH_2_ isoforms with bacterial CMs. Figure 3 shows compression isotherms for lipid monolayer representing the *P. aeruginosa* CM (black line) and the *S. aureus* CM (solid grey line) in the presence of M5-NH_2_, and those representing the *P. aeruginosa* CM (dashed black line) and the *S. aureus* CM (dashed grey line) in the presence of M5-OH (Table 2). Also shown are compression isotherms for lipid monolayers representing the *P. aeruginosa* CM (dotted black line) and the *S. aureus* CM (dotted grey line) in the absence of M5-NH_2_ isoforms. In each case, analysis of these curves was used to derive Δ*G*_*mix*_ (Table 3A), as described above (Dennison et al. 2010, 2014a)

**Fig. 4:**
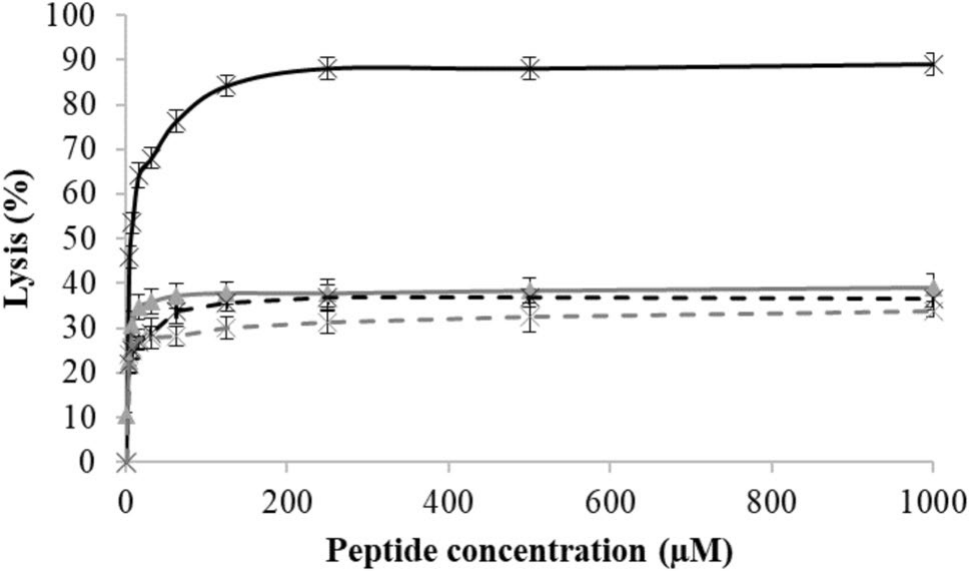
The membranolytic action of M5-NH_2_ isoforms against bacterial CMs. Figure 4 shows the change in lysis levels induced by increasing concentrations of M5-NH_2_ with calcein loaded, lipid SUVs representing the *P. aeruginosa* CM (black line) and the *S. aureus* CM (dashed black line) (Table 2). Also shown are corresponding changes in surface pressure induced by M5-OH with calcein loaded SUVs representing the *P. aeruginosa* CM (grey line) and the *S. aureus* CM (dashed grey line). In each case, maximal levels of lysis induced by peptides were determined (Table 3A) and error bars represent the standard deviation

**Fig. 5:**
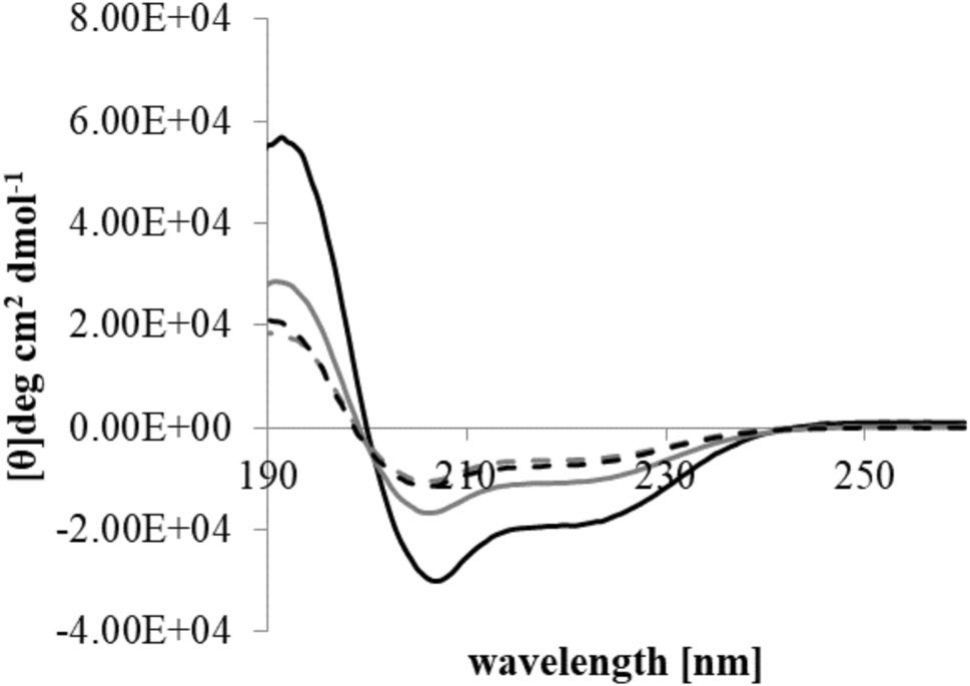
CD analysis of M5-NH_2_ isoforms in the presence of bacterial CMs. Figure 5 shows CD spectra for the conformational behaviour of M5-NH_2_ in the presence of lipid SUVs representing the *P. aeruginosa* CM, (black line) and the *S. aureus* CM, (dashed black line. Also shown are corresponding spectra for the conformational behaviour of M5-OH with lipid SUVs representing the *P. aeruginosa* CM (grey line) and *S. aureus* CM (dashed grey line) (Table 2). Minima at 208 nm and 225 nm, and maxima at 190 nm are indicative of α-helical architecture, and in each case, these spectra were analysed and levels of α-helicity determined (Table 3A), as described above (Miles et al. 2022)

In Table 3A, values of K_d_ were derived from Fig. 1; values of π were determined from Fig. 2; values of Δ*G*_*mix*_ were derived from Fig. 3; levels of lysis were obtained from Fig. 4; and levels of α-helicity were obtained from Figs. 5. In Table 3B, values of K_d_ were derived from supplementary Fig. 1; levels of α-helicity were obtained from supplementary Fig. 3; values of π were determined from supplementary Fig. 4; and levels of lysis were obtained from supplementary Fig. 5

M5-NH_2_ showed high levels of binding to SUVs mimetic of the *P. aeruginosa* CM (K_d_ = 21.5 µM; Table 3A; Fig. 1) and to characterise this affinity, the ability of the peptide to bind SUVs formed from individual CM lipids was studied (Table 2). M5-NH_2_ showed very high binding to SUVs formed from the anionic lipids of this membrane, TOCL (K_d_ = 3.7 µM) and POPG (K_d_ = 6.7 µM), but much lower binding to SUVs formed from its zwitterionic lipid, POPE (K_d_ = 14.9 µM) (Table 3B, supplementary Fig. 1) These data clearly indicated that the high affinity of M5-NH_2_ for the *P. aeruginosa* CM was primarily driven by electrostatic interactions with anionic CM lipid and complemented by minor associations with its zwitterionic lipid, which generally involves their negatively charged phosphate groups (Catte et al. 2018; von Deuster and Knecht 2012).

Compared to M5-NH_2_, the binding of M5-OH to SUVs mimetic of the *P. aeruginosa* CM were greatly decreased (K_d_ = 118.4 µM; Table 3A; Fig. 1) and represented a *circa* six-fold reduction in the affinity of M5-NH_2_ for this membrane. To investigate this reduced affinity further, the ability of M5-OH to bind SUVs formed from individual *P. aeruginosa* CM lipids was studied (Table 2). The affinity of the peptide for these SUVs followed the same rank order as for M5-NH_2_ but were at least *circa* twofold lower, namely, TOCL (K_d_ = 10.4 µM), POPG (K_d_ = 13.9 µM) and POPE (K_d_ = 26.9 µM) (Table 3B, supplementary Fig. 1). This decrease in lipid binding on C-terminal deamidation clearly suggested that the high affinity shown by M5-NH_2_ for the *P. aeruginosa* CM requires the presence of the peptide’s C-terminal amide and its associated positive charge to facilitate the electrostatic interactions involved.

M5-NH_2_, which was predominantly unstructured in aqueous solution and formed only very low levels of α-helical structure (< 5%, supplementary Fig. 2) but adopted high levels of this structure in the presence of SUVs mimetic of the *P. aeruginosa* CM (α-helicity = 80.1%; Table 3A; Fig. 5). To characterise this process, the conformational behaviour of the peptide in the presence of SUVs formed from individual lipids of the *P. aeruginosa* CM was studied (supplementary Fig. 3). M5-NH_2_ adopted very high levels of α helical structure in the presence of SUVs formed from the anionic lipids of this membrane, TOCL (α-helicity = 96.4%) and POPG** (**α-helicity = 86.1%), but much lower levels with SUVs formed from its zwitterionic lipid, POPE (α-helicity = 53.1%) (Table 3B, supplementary Fig. 3). These data clearly indicated that α-helix formation by M5-NH_2_ in the presence of the *P. aeruginosa* CM is predominantly driven by hydrophilic interactions with anionic CM lipid and complemented by minor hydrophobic interactions with its zwitterionic lipid. Given the involvement of both these types of interaction, these data also indicated that the α-helical structure formed by M5-NH_2_ possessed amphiphilic properties (Rončević, Puizina and Tossi 2019; Travkova et al. 2017).

Similarly to M5-NH_2_, M5-OH was unstructured in aqueous solution, forming only very low levels of α-helical structure (< 5%, supplementary Fig. 2), but in contrast, the levels of this structure adopted by M5-OH in the presence of SUVs mimetic of the *P. aeruginosa* CM were much lower those formed by M5-NH_2_, reduced by around a half (α-helicity = 39.6%; Table 3A; Fig. 5). To investigate this loss of α-helical structure further, the ability of M5-OH to adopt this structure in the presence of SUVs formed from individual *P. aeruginosa* CM lipids was studied (Supplementary Fig. 3). The levels of α-helicity formed by the peptide followed the same rank order as for M5-NH_2_ but were at least *circa* one third lower, namely, TOCL (α-helicity = 71.0%), POPG** (**α-helicity = 61.9%) and POPE (α-helicity = 31.1%) (Table 3B, Supplementary Fig. 3). This decrease in α-helicity on C-terminal deamidation clearly suggested that the adoption of amphiphilic α-helical structure by M5-NH_2_ at the interface of *P. aeruginosa* CM requires the presence of the peptide’s C-terminal amide. It is well-established that a major function for the C-terminal amide moiety carried by AMPs is the stabilisation of their α-helical structure, with the loss of this structural moiety leading to the formation of lower levels of this architecture by these peptides (Dennison et al. 2015; Dos Santos Cabrera et al. 2008; Sforça et al. 2004).

M5-NH_2_ showed depths of insertion into monolayer mimics of the *P. aeruginosa* CM these membranes that were indicative of penetration into their hydrophobic core region (π = 9.6 mN m^−1^; Table 3A; Fig. 2). Consistent with these data, these monolayer mimics were thermodynamically stable (Δ*G*_*mix*_ < 0; Table 2; Fig. 3) but were rendered thermodynamically unstable by interaction with M5-NH_2_ (Δ*G*_*mix*_ > 0; Table 3A; Fig. 3). This change in Δ*G*_*m*_ was consistent with increases in the lipid-packing density of these monolayers, decreases in their fluidity and deep levels of insertion by M5-NH_2_ (Dennison et al. 2010, 2014a). An ability to penetrate the hydrophobic core of bacterial membranes is generally associated with strongly membranolytic AMPs (Phoenix et al. 2013b; Zhang et al. 2021) and M5-NH_2_ showed a strong ability to permeabilize SUVs mimetic of the *P. aeruginosa* CM (lysis = 89.0%; Table 3A; Fig. 4). In combination, these data clearly indicated that M5-NH_2_ possessed potent lytic action towards the *P. aeruginosa* CM and to characterise this action, the interaction of the peptide with model membranes formed from individual CM lipids were studied (Supplementary Fig. 4). M5-NH_2_ showed strong partitioning into monolayers formed from TOCL (π = 12.3 mN m^−1^**)** and POPG (π = 9.6 mN m^−1^), but much lower insertion into for those formed from POPE (π = 4.9 mN m^−1^) (Table 3B, supplementary Fig. 4). Following the same rank order, the peptide exhibited very high levels of permeabilization with SUVs formed from TOCL (Lysis = 93.1%**)** and POPG (Lysis = 74.5%), but much lower levels with those formed from POPE (Lysis = 53.1%) (Table 3B, supplementary Fig. 5). In combination, these data indicated that the lytic action of M5-NH_2_ involved deep insertion into the *P. aeruginosa* CM that was primarily driven by electrostatic interactions with CM anionic lipid. These interactions were complemented by minor hydrophobic interactions with zwitterionic lipid, which is consistent the use of amphiphilic α-helical structure by M5-NH_2_ to promote its lytic action against the *P. aeruginosa* CM.

M5-OH also partitioned into monolayer mimics of the *P. aeruginosa* CM but these levels of insertion were around one half of those shown by M5-NH_2_ and were indicative of interactions involving the head-group and upper regions of these membranes (π = 5.1 mN m^−1^; Table 3A; Fig. 2). Reflecting this difference in insertion, these monolayer mimics remained thermodynamically stable after interaction with M5-OH (Δ*G*_*mix*_ < 0; Table 3A; Fig. 3), which is consistent with decreases in their lipid-packing density, increases in their fluidity and shallow insertion by the peptide (Dennison et al. 2010, 2014a). These data clearly indicated that the interactions of M5-OH with the *P. aeruginosa* CM differed to those of M5-NH_2,_ which was reflected in the former peptide’s ability to disrupt these membranes. M5-OH induced levels of permeabilization in SUVs mimetic of *P. aeruginosa* CM that were much lower than those of M5-NH_2_ and were reduced by *circa* one half (lysis = 39.0%; Table 3A; Fig. 4). To characterise the action of M5-OH against the *P. aeruginosa* CM, the interaction of the peptide with model membranes formed from individual CM lipids were studied (Table 2). M5-OH partitioning into monolayers formed from TOCL (π = 7.9 mN m^−1^**)** and POPG (π = 6.1 mN m^−1^) but showed much lower insertion into for those formed from POPE (π = 3.3 mN m^−1^) (Table 3B, supplementary Fig. 4). Following the same rank order, the peptide permeabilized SUVs formed from TOCL (Lysis = 60.1%**)** and POPG (Lysis = 47.5%), but at much lower levels in the case of those formed from POPE (Lysis = 32.2%) (Table 3B, Supplementary Fig. 5). These data indicated that, similarly to M5-NH_2_, the lytic action of M5-OH involved insertion into the *P. aeruginosa* CM that was primarily driven by electrostatic interactions with CM anionic lipid and complemented by minor hydrophobic interactions with its zwitterionic lipid. However, compared to M5-NH_2_, the levels of insertion and permeabilization shown by M5-OH with these individual CM lipids were decreased by around one third (Table 3B). In combination, these data clearly indicated that the lytic action of M5-NH_2_ against *P. aeruginosa* required the presence of the peptide’s C-terminal amide and its associated positive charge to facilitate the electrostatic interactions involved. These data are also consistent with a requirement for the presence of this C-terminal structural moiety to maintain the levels of amphiphilic α-helical structure adopted by M5-NH_2_ to promote its lytic action against *P. aeruginosa*.

### The M5-NH_2_ and its C-terminally deaminated isoform against *S. aureus*

As for *P. aeruginosa*, mechanisms underpinning the activity of M5-NH_2_ against *S. aureus* were investigated, and to determine any potential contribution from the peptide’s C-terminal amide moiety, this activity was compared to that of its non-amidated isoform, M5-OH. Relative to *P. aeruginosa*, M5-NH_2_ showed activity against *S. aureus* (MLC = 139.6 µM; Table 3A) that was over 20-fold lower, clearly indicating that *S. aureus* was strongly resistant to the peptide. A number of mechanisms are used by *S. aureus* to resist AMPs and to determine those potentially involved in the case of M5-NH_2_, the interaction of the peptide with the organism’s CM was studied (Assoni et al. 2020; Cheung et al. 2018; Joo and Otto 2015).

M5-NH_2_ showed an ability to bind SUVs mimetic of these membranes (K_d_ = 120.6 µM; Table 3A; Fig. 1) that was around fivefold weaker than that in the case of the *P. aeruginosa* CM, indicating that the mechanism used by *S. aureus* to resist M5-NH_2_ had effectively lowered the peptide’s affinity for the organism’s CM. To characterise the affinity of M5-NH_2_ for the *S. aureus* CM, the ability of the peptide to bind individual CM lipids was considered, namely, TOCL, POPG and Lys-PG (Table 2). TOCL and POPG represent the major anionic lipids in the *S. aureus* CM and would strongly promote the binding of M5-NH_2_ to the *S. aureus* CM, given the peptide’s very high affinity for these lipids (K_d_ < 7.0 µM, Table 3B, supplementary Fig. 1). Lys-PG is a cationic lipid, and it has been predicted that the presence of Lys-PG in the *S. aureus* CM can promote electrostatic repulsion effects that reduce the overall affinity of M5-NH_2_ for this membrane (Dennison et al. 2023). This prediction could not be tested directly due the labile nature of Lys-PG and the instability of model membranes formed from this lipid alone (Wölk et al. 2020). However, this prediction was strongly supported by results indicating that M5-NH_2_ showed no interaction with monolayer mimics of the *S. aureus* CM where anionic lipid had been replaced by corresponding levels of zwitterionic lipid, thus exposing the peptide solely to the intrinsic positive charge of this membrane (data not shown).

M5-OH exhibited levels of activity against *S. aureus* that were highly comparable to those of M5-NH_2_ (MLC = 133.3 µM; Table 3A), which is consistent with other studies and showed C-terminal deamidation to have no significant effect on the levels of resistance shown by the organism to the peptide (Owen 2005). The reasons for this effect were unclear, but clearly, the decreased positive charge associated with C-terminal deamidation will affect the electrostatic repulsion effects experienced by M5-NH_2_ and hence, its level of binding to the *S. aureus* CM, as for other AMPs (da Silva et al. 2014; Zhu et al. 2021). However, this effect was not apparent, evidenced by the fact that the ability of M5-OH to bind the *S. aureus* CM was similar to that of M5-NH_2_ (K_d_ = 115.8 µM; Table 3A; Fig. 1). As in the case of M5-NH_2_, M5-OH also showed no interaction with monolayer mimics of the *S. aureus* CM where anionic lipid had been replaced by corresponding levels of zwitterionic lipid. In combination, these results suggested that Lys-PG-mediated electrostatic repulsion effects had not been appreciably affected by the decrease in positive charge of M5-NH_2_, with the overall result that its affinity for the *S. aureus* CM was essentially unchanged by loss of its C-terminal amide group.

Although the affinity of M5-NH_2_ for the *S. aureus* CM was greatly reduced in comparison to that of the *P. aeruginosa* CM, this reduced affinity appeared to limit rather than abolish the peptide’s ability to access the former membrane (Table 3A, Fig. 2). In the presence of SUVs mimetic of the *S. aureus* CM, M5-NH_2_ adopted amphiphilic α-helical structure that were in the region of one third of those shown in the case of the *P. aeruginosa* CM (α-helicity = 30.1%; Table 3A; Fig. 5). TOCL and POPG would strongly promote the formation of this structure by M5-NH_2_, as demonstrated for the peptide in the case of the *P. aeruginosa* CM (α-helicity > 85%, Table 3B, supplementary Fig. 3). However, the low levels of amphiphilic α-helical structure adopted by M5-NH_2_ in the presence of the *S. aureus* CM would be consistent with the involvement of the Lys-PG-mediated defence mechanism suggested above to play a role in the resistance of *S. aureus* to M5-NH_2_. According to this mechanism, these low levels of α-helicity would result from electrostatic repulsion effects between the peptide and Lys-PG in the *S. aureus* CM that effectively raise the energy barrier for α-helix formation by the peptide, as in the case of other AMPs (Rehal et al. 2021; Simcock et al. 2020). As described above, loss of the α-helix stabilisation provided by the C-terminal amide of M5-NH_2_ would promote changes to the levels of α-helical structure formed by the peptide, as shown for other AMPs (Dennison et al. 2015; Mura et al. 2016; Pozo Ramajo et al. 2005; Sforça et al. 2004). However, this effect was not apparent, evidenced by the fact that M5-OH adopted levels of amphiphilic α-helical structure that were similar to those of M5-NH_2_ (α-helicity = 29.8%; Table 3A; Fig. 5). These results suggested that conformational changes associated with C-terminal deamidation were negated by the high energy barrier posed by Lys-PG-mediated electrostatic repulsion effects, with the overall result that the levels of α-helicity formed by M5-NH_2_ were essentially unchanged by loss of its C-terminal amide group.

M5-NH_2_ showed levels of penetration into monolayer mimics of the *S. aure*us CM that were around half of those shown with the *P. aeruginosa* CM (π = 4.8 mN m^−1^; Table 3A; Fig. 2) and indicative of interactions with the head-group and upper regions of the *S. aureus* CM (Dennison et al. 2010, 2014a). Consistent with these low levels of penetration, these monolayer mimics showed thermodynamic stability that was retained after interaction with M5-NH_2_ (Δ*G*_*mix*_ < 0; Table 3A; Fig. 3), contrasting strongly to the corresponding case with the *P. aeruginosa* CM (Table 3A). As described above, the retention of Δ*G*_*mix*_ values < 0 is indicative of changes to properties of the *S. aureus* CM that are associated with shallow insertion by M5-NH_2_ rather than the deep levels of penetration shown by the peptide with the *P. aeruginosa* CM (Table 3A) (Dennison et al. 2010, 2014a). M5-NH_2_ also showed a weak ability to permeabilise SUVs mimetic of the *S. aure*us CM, achieving levels that were *circa* one third of those shown by the peptide in the case of the *P. aeruginosa* CM (36.4%; Table 3A; Fig. 4) In combination, these data strongly suggested that Lys-PG-mediated reductions in the ability of M5-NH_2_ to bind the *S. aure*us CM and adopt amphiphilic α-helical structure decreased the lytic action of the peptide against *S. aure*us, as compared to that towards *P. aeruginosa*.

M5-OH penetrated monolayer mimics of the *S. aure*us CM at levels (π = 4.1 mN m^−1^; Table 3A; Fig. 2) that were comparable to those of M5-NH_2_ (Table 2). Showing further similarities to M5-NH_2_, these monolayer mimics also showed thermodynamic stability that was retained after interaction with M5-OH (Δ*G*_*mix*_ < 0; Table 3A; Fig. 3), indicating shallow insertion of the peptide into the *S. aure*us CM (Dennison et al. 2010, 2014a). These similarities were maintained in the ability of M5-OH to permeabilize SUVs mimetic of this membrane (lysis = 33.7%; Table 3A; Fig. 5), which showed levels of lysis that were close to those shown by M5-NH_2_ (Table 3A). In combination, these data clearly suggested that loss of the C-terminal amide carried by M5-NH_2_ had not significantly impacted on the peptide’s lytic action against *S. aure*us.

## Discussion

The incorporation of a C-terminal amide moiety into synthetic α-helical AMPs has produced a number of antibacterial peptides that have been clinically trialled, including pexiganan, described above (Dijksteel et al. 2021), and more recently, PL-5 for the treatment of skin infections (Li et al. 2017b). This PTM is also a structural feature of the designed α-helical peptide, M5-NH_2_ (Owen 2005), recently shown to be essential for the activity of the peptide against *E. coli* (Dennison and Phoenix 2011a; Tanaka et al. 2023), which is classed as a critical-priority pathogen (World Health Organization 2024). The present study investigates the potential role of C-terminal amidation in the activity of M5-NH_2_ against *P. aeruginosa* and *S. aureus*, which are currently classed as having high priority within the world’s most dangerous bacterial pathogens (World Health Organization 2024).

M5-NH_2_ killed *P. aeruginosa* at levels in the low micromolar range, indicating potent activity against the organism and the ability to pass through the outer layer of the OM of the organism. It has previously been observed that the high molecular weight of M5-NH_2_ renders it unlikely that the peptide would achieve this passage by diffusion through porins and self-promoted uptake appeared to be the pathway most favoured by strongly cationic peptides such as M5-NH_2_ (Dennison et al. 2023). Loss of the C-terminal amide moiety possessed by M5-NH_2_ reduced its activity against *P. aeruginosa* to levels that effectively rendered the peptide ineffective against the organism, clearly showing that this C-terminal moiety played a functional role in this activity (Table 3A). One major effect of its removal would be to decrease the positive charge of M5-NH_2,_ suggesting that the peptide’s native charge (Table 1) may be the threshold for activity against *P. aeruginosa* and lowering this charge below this threshold through C-terminal deamination led to the observed loss of antibacterial activity (Dennison et al. 2009; Owen 2005). These observations are consistent with other studies (Dennison and Phoenix 2011a), and it is generally accepted that varying the positive charge of AMPs outside of certain limits can promote undesirable side effects, including a loss of antibacterial efficacy (Kumar et al. 2018). This threshold is often reflected as a minimum charge requirement for the efficient electrostatic binding of AMPs to anionic lipid in the bacterial CM, which appeared to be the case for M5-NH_2_ (Preußke et al. 2023).

In this early step of its action against *P. aeruginosa*, the peptide showed high levels of binding to the organism’s CM that were in the low micromolar range, which is consistent with previous work and indicated a strong affinity for these membranes (Dennison and Phoenix 2011a; Dennison et al. 2019). M5-NH_2_ also showed a strong preference for binding the major anionic lipids found in the *P. aeruginosa* CM, as compared to its zwitterionic lipid (Table 3B). This preference is known to involve electrostatic interactions between the headgroups of these anionic lipids and the peptide’s cationic residues, namely, lysine’s and a C-terminally amidated leucine (Bessalle et al. 1993; Dennison et al. 2012). However, on C-terminal deamidation, the levels of binding shown by M5-NH_2_ with the *P. aeruginosa* CM were reduced by orders of magnitude and although the peptide retained a strong preference for anionic lipid, its affinity for this lipid was greatly reduced (Table 3A, B). These observations clearly suggested that the decreased charge on M5-NH_2_ due to this structural change contributed to the peptide’s loss of activity against *P. aeruginosa* by lowering its affinity for anionic lipid in the organism’s CM (Table 3A). Very recent studies have suggested the possibility that other chemical properties of the C-terminal amide moiety carried by M5-NH_2_ may also contribute to the affinity of the peptides for bacterial membranes (Shahmiri and Mechler 2020). In this work, the primary amine of C-terminal amides carried by amphibian AMPs was substituted with a secondary amine, effectively maintaining its positive charge but changing its hydration state by reducing its hydrogen bond provision and water access (Kuemin et al. 2009; Shahmiri and Mechler 2020). These structurally modified peptides showed a greatly reduced affinity for membranes, which led to the proposal that the C-terminal amide of some AMPs may play a role as a specific binding motif, where its hydration state helps facilitate the entry of these peptides into the bilayer headgroup region (Shahmiri and Mechler 2020).

In the later stages of its action against *P. aeruginosa*, M5-NH_2_ adopted high levels of amphiphilic α-helical structure that essentially drove insertion into the hydrophobic core of the organism’s CM and promoted high levels of lysis. However, loss of the C-terminal moiety carried by M5-NH_2_ greatly reduced these levels of amphiphilic α-helix formation and membrane interaction, clearly indicating that this moiety was required for the later stages of the peptides action against *P. aeruginosa*. These observations are consistent with another major effect associated with the C-terminal deamidation of α-helical AMPs: the additional hydrogen bonding interactions provided by this structural moiety (Kuemin et al. 2009; Shi et al. 2001) is well known to promote membranolytic action by enhancing the rigidity, stability and length of their structure (da Silva et al. 2014; Mura et al. 2016). In the case of M5-NH_2_, this α-helical structure involved around three quarters of the peptide’s residues (Table 3A), which equated to just over three α-helical turns and is sufficient to span a membrane leaflet (Rončević et al. 2019). The amphiphilicity of α-helical AMPs is characterised by a segregation of hydrophilic and hydrophobic residues about the α-helical long axis that allows these peptides to engage in concomitant interactions with the polar and apolar compartments of the bacterial CM (Li et al. 2021; Zhang et al. 2021). In the case of M5-NH_2_, this amphiphilicity would allow the peptide to engage in stabilising electrostatic interactions with the headgroups of anionic lipid in the *P. aeruginosa* CM and insert into the hydrophobic core of this membrane, thereby inducing the observed changes to the mechanical properties of the CM that together promote lysis (Table 3A) (Dennison et al. 2010, 2014a). These changes included decreases in fluidity and increases in lipid-packing density (Table 3A), and similar effects have been reported for the membranolytic action of other AMPs which appear to result from the general ability of these peptides to order and reduce the lateral motion of lipids when inserted into the bacterial CM (Morales-Martínez et al. 2022; Qian and Zolnierczuk 2022). Taken in combination, these observations would support a toroidal-type pore mechanism, which has been proposed likely to describe the membranolytic action of M5-NH_2_ against *P. aeruginosa* (Dennison et al. 2023). Essentially, using this mechanism, molecules of M5-NH_2_ would induce insert deeply into the *P. aeruginosa* CM and promote lysis by inducing a continuous bending of this membrane until transmembrane channels formed through the intercalation of reorientated M5-MH_2_ molecules with lipid (Rončević et al. 2019; Wang et al. 2023).

In contrast to M5-NH_2_, the peptide’s C-terminally deamidated isoform adopted levels of amphiphilic α-helical structure that accounted for one third of its sequence (Table 3A) and equated to *circa* one and a half α-helical turns (Rončević et al. 2019). Recent work on a number of AMPs has demonstrated that loss of the hydrogen bonding and α-helix stabilisation provided by this PTM leads to the partial unravelling and loss of α-helical structure, primarily due to reduced steric restrictions (Dennison et al. 2015; Mura et al. 2016; Pozo Ramajo et al. 2005; Sforça et al. 2004). The length of amphiphilic α-helical structure formed by AMPs correlates with their depths of membrane penetration (Gagnon et al. 2017; Li et al. 2021) and the C-terminally deamidated isoform of M5-NH_2_ showed greatly reduced penetration of the *P. aeruginosa* CM relative to the native peptide (Table 3A). Consistent with its lower levels of amphiphilic α-helical structure, this isoform induced only low levels of lysis in the *P. aeruginosa* CM, which involved shallow insertion and association with its upper regions rather than deeply penetrating its hydrophobic core, as with M5-NH_2_ (Table 3A) (Dennison et al. 2010, 2014a; Rončević, Puizina and Tossi 2019). The insertion of the peptide into the *P. aeruginosa* CM also appeared to show major differences to that of native M5-NH_2_ in its effect on mechanical properties of these membranes (Table 3A), inducing decreases in their lipid-packing density and increases in their fluidity (Dennison et al. 2010, 2014a). These effects have been described for other AMPs and have been primarily ascribed to membrane expansion through the insertion of these peptides into CM lipid headgroup region, which can lead to decreased ordering and increased lateral motion of its lipids (Dennison et al. 2006, 2007, 2008). Taken in combination, these observations suggest that the membranolytic action of C-terminally deamidated M5-NH_2_ against *P. aeruginosa* may involve early stages in a carpet-type mechanism. Using this mechanism, the peptide would promote lysis of the *P. aeruginosa* CM via a detergent-like action that involves the induction of leaky, transient surface lesions in these membranes rather than full scale lysis (Phoenix, Dennison and Harris 2013b; Zhang et al. 2021).

*S. aureus* was clearly resistant to the action of the M5-NH_2_, evidenced by the levels of the peptide that were required to kill the organism, which were orders of magnitude higher than those required in the case of *P. aeruginosa* (Table 3A). The peptide’s C-terminally deamidated isoform killed *S. aureus* at similar levels, indicating that this structural modification had not appreciably affected the overall level of resistance shown by the organism to the action of M5-NH_2_ (Table 3A). It is well established that C-terminal deamidation can have a variable influence the antibacterial efficacy of AMPs and to gain further insight into these observations, mechanisms underpinning the resistance of *S. aureus* to these M5-NH_2_ isoforms were investigated (Dennison et al. 2009). These mechanisms appeared to be primarily mediated by Lys-PG, which is enzymatically generated from PG in the inner leaflet of the *S. aureus* CM and then translocated to the outer leaflet of this membrane (Sohlenkamp and Geiger 2015). Lys-PG is the only known cationic, bacterial lipid (Sohlenkamp and Geiger 2015) and it is generally believed that the lipid is evenly distributed within the anionic lipid matrix of the *S. aureus* CM, predominantly formed from PG and CL (Ernst and Peschel 2011). Taken with its high occurrence in these membranes, these properties allow Lys-PG to promote effects that induce resistance to AMPs by inhibiting key steps in the action of these peptides, namely, binding and α-helix formation (Assoni et al. 2020; Shireen et al. 2013).

Lys-PG appeared to inhibit the binding of M5-NH_2_ to the *S. aureus* CM, with the peptide displaying an affinity for this membrane that was many-fold lower than that in the case of *P. aeruginosa* CM (Table 3A). Similarly, the ability of this lipid to inhibit the formation of α-helical structure by M5-NH_2_ with the *S. aureus* CM was indicated by the fact that the levels of this structure were less than one third of those in the case of *P. aeruginosa* (Table 3A). These decreased levels of affinity and α-helix formation appeared to result from the high level of electrostatic repulsion effects between the peptide’s cationic lysine and amidated leucine residues and the positively charged head group carried by Lys-PG. These electrostatic effects appeared to pose a barrier that repelled M5-NH_2_ from the *S. aureus* CM, thereby inhibiting binding, and raised the energy threshold for conformational changes involved in α-helix formation, thereby inhibiting the peptide’s ability to adopt this structure. Results obtained elsewhere and above for *P. aeruginosa*, clearly showed that C-terminal deamidation can affect the interactions of M5-NH_2_ with the bacterial CM, which suggested the possibility that this could also be the case for *S. aureus* CM (Dennison and Phoenix 2011a; Tanaka et al. 2023). The decrease in positive charge associated with the C-terminal deamidation of M5-NH_2_ will reduce the electrostatic repulsion effects experienced by the peptide, thereby potentially promoting changes to its affinity for the *S. aureus* CM (Gan et al. 2021). Loss of the hydrogen bonding and α-helix stabilisation provided by the C-terminal amide of moiety of M5-NH_2_ will also potentially promote changes to the levels of amphiphilic α-helical structure formed by the peptide in the presence of the *S. aureus* CM (Gan et al. 2021). However, the affinity of C-terminally deamidated M5-NH_2_ for the *S. aureus* CM and the levels of α-helical structure adopted by the peptide in the presence of this membrane were similar to those of M5- NH_2_ (Table 3A). These observations indicated that changes to the interactions of M5-NH_2_ with the *S. aureus* CM due to C-terminal deamidation were either rendered insignificant or negated by the strong Lys-PG-mediated electrostatic repulsion effects experienced by the peptide.

Clearly, the levels of charge carried by M5-NH_2_ and its C-terminally deamidated isoform played a key role in promoting the Lys-PG-mediated inhibition of CM affinity and α-helix formation involved in the resistance of *S. aureus* to their action (Table 3A). Similar results has been reported for CP7 and AMH (Simcock et al. 2020), which are synthetic α-helical AMPs, with comparable levels of cationicity to the M5-NH_2_ isoforms studied here (Table 1) (Hammond et al. 2019; Rakowska et al. 2013; Simcock et al. 2020). However, a role for other charge-related properties in the Lys-PG-mediated resistance of *S. aureus* to AMH and CP7 has been suggested, including a high charge density and its sequence distribution; characteristics well known to modulate the antibacterial efficacy of AMPs (Hellewell et al. 2022). AMH and CP7 possess charge densities that are similar to those of the M5-NH_2_ isoforms studied here (> 0.35, Table 1) and are generally much greater than those of most natural, α-helical AMPs with activity against *S. aureus* (< 0.25, Table 4). Moreover, the cationic residues responsible for the high charge density of AMH and CP7 display an even distribution along their sequences that is similar to that of M5-NH_2_ and its C-terminally deamidated isoform (Table 3). The high magnitude and regular distribution of charge density appears to maximise the potential of AMH and CP7 to encounter Lys-PG-mediated electrostatic repulsion effects underpinning *S. aureus* resistance to their action, suggesting a similar role in the case of the M5-NH_2_ isoforms studied here (Simcock et al. 2020). Indeed, it would seem that the possession of these charge-related properties could help explain the fact that *S. aureus* is able to resist some AMPs with similar levels of charge to CP7, AMH and M5-NH_2_ isoforms (Table 1) but that the organism is highly susceptible to the action of others (Ciandrini et al. 2018; Zouhir et al. 2016).

**Table 4:** Charge-based characteristics of α-helical AMPs with activity against *S. aureus*

AMPs	Charge	Charge density	MLC (µM)
Taac-CATH1	+ 8	0.22	3.9
pxCECA1	+ 8	0.19	1.8
Cramp-2	+ 7	0.18	32.0
BMAP-28	+ 7	0.24	2.0
LL-37	+ 6	0.16	4.0
Cramp-1	+ 6	0.18	32.0
Esculentin-2B	+ 5	0.14	1.0
Brevinin-2PRe	+ 5	0.14	25.0
Brevinin-1AUa	+ 4	0.17	3.0
XT-1	+ 4	0.15	5.0
Magainin 2	+ 3	0.13	18.0
Ranalexin-1Ca	+ 3	0.15	17.0
Hylaseptin-P1	+ 2	0.15	6.1
Kassinatuerin-1	+ 2	0.10	8.0
Caerin-1.1	+ 1	0.04	3.0
Aurein-1.2	+ 1	0.08	17.0

Table 4 shows the charge, charge density and MLC for representative naturally occurring, α-helical AMPs with activity against *S. aureus*. The charge density of these AMPs is defined as their average net charge per residue and activity against *S. aureus* is taken as an MLC (minimum lethal concentration) of less than 50.0 µM, as described in (Islam et al. 2023; Zouhir et al. 2016). Data for these AMPs were derived from (Islam et al. 2023; Wang, Li and Wang 2016; Zouhir et al. 2016)

Although *S. aureus* was strongly resistant to the M5-NH_2_ isoforms studied here, these peptides showed some ability to access the *S. aureus* CM that was presumably transient and driven by their high affinity for the anionic lipid in these membranes. In the case of both peptides, this access resulted in weak membranolytic action against the *S. aureus* CM that was promoted by levels of α-helicity accounting for *circa* one quarter of their structure, which is around one α-helical turn (Rončević, Puizina and Tossi 2019). These levels of α-helicity would predict interaction with the headgroup region of the *S. aureus* CM, which was strongly supported by the ability of these peptides to induce decreases in the lipid-packing density and increases the fluidity of this membrane (Table 3A) (Dennison et al. 2010, 2014a; Im and Brooks 2005). These CM structural changes were accompanied by low levels of penetration and lysis, which was consistent with the weak membranolytic action observed here for M5-NH_2_ and its C-terminally deamidated isoform towards the *S. aureus* CM (Table 3A). In this action, the levels of amphiphilic α-helical structure, CM interaction and effects on CM structure shown by these peptides were comparable to those exhibited by C-terminally deamidated M5-NH_2_ with the *P. aeruginosa* CM (Table 3A). These observations clearly suggested that the weak membranolytic action of the M5-NH_2_ isoforms studied here towards *S. aureus* may involve a carpet-type mechanism with general similarities to that proposed above for C-terminally deamidated M5-NH_2_ against *P. aeruginosa*. However, it also seems likely that there could be differences between these mechanisms given the widely different lipid compositions of the *S. aureus* and *P. aeruginosa* CM (Table 2). For example, Lys-PG is absent from the *P. aeruginosa* CM and indirect membrane effects due to the lipid could contribute to the weak membranolytic action of the M5-NH_2_ isoforms studied here against *S. aureus*. It has previously been demonstrated that the ability of the lipid to reduce electrostatic repulsion between the headgroups of anionic lipids leads to decreases the fluidity of the *S. aureus* CM that make partitioning by AMPs more difficult (Cox et al. 2014).

## Conclusions

Here, M5-NH_2_ was investigated for its activity against *P. aeruginosa* and *S. aureus* and the effect of C-terminal deamidation on this activity, which revealed strongly contrasting results. The high level of charge possessed by the peptide promoted the death of *P. aeruginosa* through potent membranolytic action but was rendered ineffective against the organism by C-terminal deamidation and its inhibitory effect on key steps in this action. Taken with other work, these results clearly suggested that the positive charge and α-helix stabilising ability of the C-terminal amide carried by M5-NH_2_ is a general requirement for its activity against Gram-negative bacteria (Dennison et al. 2023; Dennison and Phoenix 2011c). Based on these observations, attempts to improve the antibacterial activity of M5-NH_2_ have shown that the conversion of its C-terminal amide to a hydrazine group enhanced its activity against *E. coli* (Tanaka et al. 2023). This chemical modification had no significant effect on the positive charge of the parent amide moiety, and it was suggested that the C-terminal hydrazidation of AMPs enhances their action against *E. coli*, *P. aeruginosa* and other bacteria through increased resistance to proteolysis by bacterial enzymes (Li et al. 2017c; Tanaka et al. 2023). However, the membranolytic action of M5-NH_2_ appears to involve tilted insertion into the bacterial CM with its amidated C-terminus located in the membrane’s hydrophobic core, which is energetically unfavourable (Dennison et al. 2019, 2023; Dennison and Phoenix 2011c). We speculate that the introduction of a hydrazine group may increase the overall hydrophobicity of the peptide’s C-terminus, thereby increasing the efficacy of its penetration into the *E. coli* CM and its activity against the organism. Given the potential of M5-NH_2_ for development as an agent to combat infections due to *P. aeruginosa*, it may be fruitful to investigate the effect of a similar C-terminal substitution on the activity of the peptide against this organism (Dennison et al. 2023).

In contrast to *P. aeruginosa*, the high level of charge possessed by M5-NH_2_ facilitated *S. aureus* resistance to the peptide’s membranolytic action through Lys-PG-mediated mechanisms and their inhibitory effects on key steps in this action. In further contrast to *P. aeruginosa*, loss of the positive charge and α-helix stabilising ability of the C-terminal amide carried by M5-NH_2_ had no discernible effect on either Lys-PG-mediated inhibitory effects or the levels of resistance shown by *S. aureus* to the action of the peptide. Comparisons to other AMPs suggested that *S. aureus* resistance to the M5-NH_2_ isoforms studied here may depend not only on the level of charge possessed by these peptides but also its density and distribution along their sequences (Table 1). The possession of these charge-related properties go some way towards helping explain the differing efficacies of AMPs against the Lys-PG-mediated mechanisms used by *S. aureus* to resist the action of these peptides. However, ovispirin-1 is an ovine, α-helical peptide that has a comparable level, density and distribution of charge to those of M5-NH_2_ and might therefore be predicted to inactive against *S. aureus* (Jiang et al. 2008) but, instead, exhibits potent activity against the organism (Sawai et al. 2002). These observation clearly indicate that charge-related properties of AMPs alone do not necessarily determine whether *S. aureus* is susceptible to these peptides or able to resist their action using Lys-PG-mediated mechanisms. Indeed, this difference can be triggered by a single change of one uncharged residue for another, as recently demonstrated for NK-2, which is a mammalian α-helical peptide with comparable charge properties to M5-NH_2_ (Andrä et al. 2011). Although charge-related factors that promote *S. aureus* resistance to AMPs have been identified as common to subsets of these peptides, in general, peptide-based factors involved in this resistance are not well characterised, and no universal example appears to be known.

Clearly, the ability of *S. aureus* to resist the M5-NH_2_ isoforms studied here also depends upon the high level of Lys-PG in the organism’s CM and the role of this lipid in the resistance of *S. aureus* to AMPs is well described (Ernst and Peschel 2011; Miller, Bayer and Arias 2016). In relation to the M5-NH_2_ isoforms studied here, recent work showed levels of Lys-PG found in the CM of the organism to be optimal for defence against their action (Dennison et al. 2023). This work also predicted that in the case of other bacteria, resistance to the M5-NH_2_ isoforms studied here was likely to require levels of Lys-PG in the CM of these organisms that was of the order of those found in the case of *S. aureus* (Dennison et al. 2023). Consistent with this suggestion, the CM of *Bacillus subtilis* has a Lys-PG content of around one half that of *S. aureus* (Roy 2009) and M5-NH_2_ showed potent membranolytic action against the organism (Dennison et al. 2019). Currently, an increasing number of bacteria that include Lys-PG in their CM are being reported, which could potentially provide further insight into the use of mechanisms based on the lipid by both *S. aureus* and other bacteria to resist the M5-NH_2_ isoforms studied here (Joyce and Doran 2023; Slavetinsky, Kuhn and Peschel 2017; Sohlenkamp and Geiger 2015). However, a clear implication from these observations is that the antibacterial efficacy of M5-NH_2_ and other AMPs can depend upon not only the occurrence a given lipid in the bacterial CM but also the levels of that lipid present. Analogous examples are known for other bacterial lipids; for example, PE is known to promote the resistance of bacteria to the amphibian peptide, maximin H5, and to promote the antibacterial action of cyclotides, which are AMPs from plants (Phoenix et al. 2015).

In summary these results have provided a more detailed description of mechanisms involved in the susceptibility and resistance of bacteria to M5-NH_2_ and have emphasised the importance of a holistic view when designing synthetic AMPs. In this design, it is essential to consider not only the characteristics of these peptides, such as charge-related properties, but also those of their target bacterial membranes, and in particular, their lipid composition.

## Supplementary Information

Below is the link to the electronic supplementary material.

Supplementary file1 Supplementary Figure 1. The binding of M5-NH_2_ isoforms to individual CM lipids. Supplementary Figure 1 shows the change in fluorescence (ΔF) induced by increasing concentrations of M5-NH_2_ with FPE-labelled SUVs formed from the individual CM lipids: TOCL (black), POPG (dark grey) and POPE (light grey). Also shown are corresponding changes in fluorescence induced by M5-OH with FPE-labelled SUVs formed from TOCL (dotted black), POPG (dotted dark grey) and POPE (dotted light grey). In each case, analysis of these curves was used to derive K_d_ (Table 3B), as described above, and error bars represent the standard deviation (Maman 2022). (TIF 67 KB)

Supplementary file2 Supplementary Figure 2. CD analysis of M5-NH_2_ isoforms in aqueous solution. Supplementary Figure 2 shows CD spectra for the conformational behaviour of M5-NH_2_ (black) and M5-OH (grey) in aqueous solution. Maxima at 215 nm and minima at 198 nm is indicative of random coil and β-type structures, and in each case, these spectra were analysed and levels of secondary structure determined (Table 3B), as described above (Miles, Ramalli and Wallace 2022).(TIF 56 KB)

Supplementary file3 Supplementary Figure 3. CD analysis of M5-NH_2_ isoforms in the presence of individual CM lipids. Supplementary Figure 3 shows CD spectra for the conformational behaviour of M5-NH_2_ in the presence of SUVs formed from TOCL (black), POPG (dark grey) and POPE (light grey). Also shown are corresponding spectra for the conformational behaviour of M5-OH with SUVs formed from TOCL (dotted black), POPG (dotted dark grey) and POPE (dotted light grey). Minima at 208 nm and 225 nm, and maxima at 190 nm are indicative of α-helical architecture, and in each case, these spectra were analysed and levels of α-helicity determined (Table 3B), as described above (Miles, Ramalli and Wallace 2022).(TIF 57 KB)

Supplementary file4 Supplementary Figure 4. The interaction of M5-NH_2_ isoforms with individual CM lipids. Figure 4 shows the change in surface pressure induced by increasing concentrations of M5-NH_2_ in lipid monolayers formed from solid lines TOCL (black), POPG (dark grey) and POPE (light grey). Also shown are corresponding changes in surface pressure induced by M5-OH with monolayers formed from TOCL (dotted black), POPG (dotted dark grey) and POPE (dotted light grey). In each case, maximal surface pressures were determined (Table 3B).(TIF 53 KB)

Supplementary file5 Supplementary Figure 5. The membranolytic action of M5-NH_2_ isoforms against individual CM lipids. Supplementary Figure 5 shows the change in lysis levels induced by increasing concentrations of M5-NH_2_ with calcein loaded, SUVs formed from TOCL (black), POPG (dark grey) and POPE (light grey). Also shown are corresponding changes in surface pressure induced by M5-OH with calcein loaded SUVs formed from TOCL (dotted black), POPG (dotted dark grey) and POPE dotted light grey). In each case, maximal levels of lysis induced by peptides were determined (Table 3B) and error bars represent the standard deviation. (TIF 57 KB)

## Data Availability

All data supporting the findings of this study are available within the paper.

## Abbreviations

AMPs: Antimicrobial peptides
CL (TOCL, 1,1',2,2'-tetraoleoyl cardiolipin): 1',3'-Bis[1,2-dioleoyl-sn-glycero-3-phospho]-glycerol.
CM: Cytoplasmic membrane
EDTA: Ethylenediaminetetraacetic acid.
HEPES: M 4-(2-hydroxyethyl)-1-piperazineethanesulfonic acid.
HPLC: High-performance liquid chromatography.
Lys-PG (Lys-DOPG): 1,2-Dioleoyl-sn-glycero-3-[phospho-rac-(3-lysyl(1-glycerol))].
MLC: Minimum lethal concentration
M5-NH_2_: Modelin-5
M5-OH: C-terminally deamidated modelin-5.
PBS: Phosphate buffered saline.
POPC: 1-Palmitoyl-2-oleoyl-sn-glycero-3-phosphocholine.
POPE: 1-Palmitoyl-2-oleoyl-sn-glycero-3-phosphoethanolamine.
POPG: 1-Palmitoyl-2-oleoyl-sn-glycero-3-phosphoglycerol.
SUVs: Small unilamellar vesicles
