# Supplementary figures and images for: The effect of C-terminal deamidation on bacterial susceptibility and resistance to modelin-5
Source: Eur Biophys J. 2025 Feb 11;54(1-2):45–63. doi: 10.1007/s00249-025-01732-4 (PMC11880157; doi:10.1007/s00249-025-01732-4)

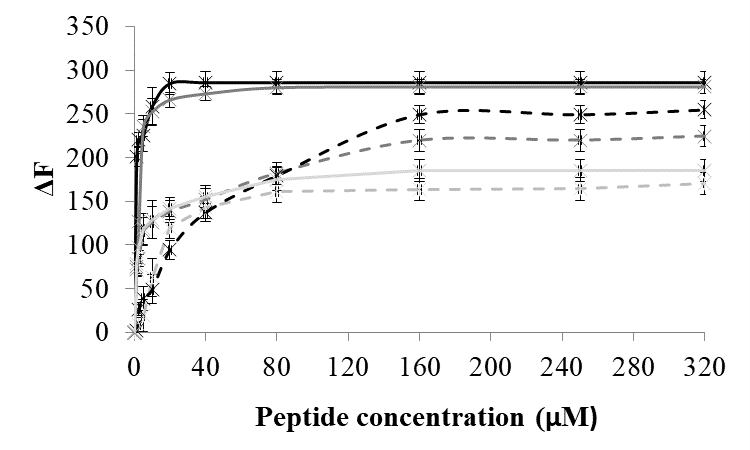

Supplement: Supplementary file 1 — Supplementary file1 Supplementary Figure 1. The binding of M5-NH2 isoforms to individual CM lipids. Supplementary Figure 1 shows the change in fluorescence (ΔF) induced by increasing concentrations of M5-NH2 with FPE-labelled SUVs formed from the individual CM lipids: TOCL (black), POPG (dark grey) and POPE (light grey). Also shown are corresponding changes in fluorescence induced by M5-OH with FPE-labelled SUVs formed from TOCL (dotted black), POPG (dotted dark grey) and POPE (dotted light grey). In each case, analysis of these curves was used to derive Kd (Table 3B), as described above, and error bars represent the standard deviation (Maman 2022). (TIF 67 KB) [file 249_2025_1732_MOESM1_ESM.tif]

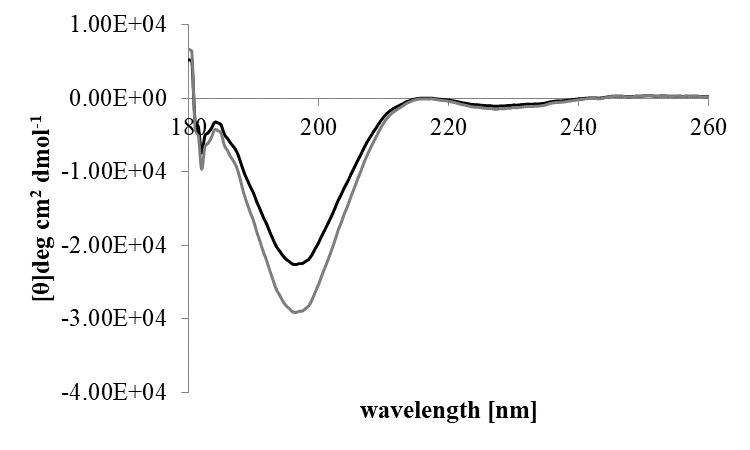

Supplement: Supplementary file 2 — Supplementary file2 Supplementary Figure 2. CD analysis of M5-NH2 isoforms in aqueous solution. Supplementary Figure 2 shows CD spectra for the conformational behaviour of M5-NH2 (black) and M5-OH (grey) in aqueous solution. Maxima at 215 nm and minima at 198 nm is indicative of random coil and β-type structures, and in each case, these spectra were analysed and levels of secondary structure determined (Table 3B), as described above (Miles, Ramalli and Wallace 2022).(TIF 56 KB) [file 249_2025_1732_MOESM2_ESM.tif]

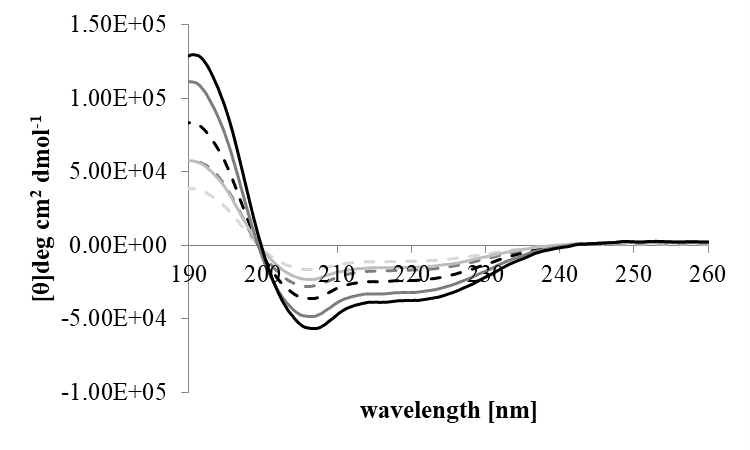

Supplement: Supplementary file 3 — Supplementary file3 Supplementary Figure 3. CD analysis of M5-NH2 isoforms in the presence of individual CM lipids. Supplementary Figure 3 shows CD spectra for the conformational behaviour of M5-NH2 in the presence of SUVs formed from TOCL (black), POPG (dark grey) and POPE (light grey). Also shown are corresponding spectra for the conformational behaviour of M5-OH with SUVs formed from TOCL (dotted black), POPG (dotted dark grey) and POPE (dotted light grey). Minima at 208 nm and 225 nm, and maxima at 190 nm are indicative of α-helical architecture, and in each case, these spectra were analysed and levels of α-helicity determined (Table 3B), as described above (Miles, Ramalli and Wallace 2022).(TIF 57 KB) [file 249_2025_1732_MOESM3_ESM.tif]

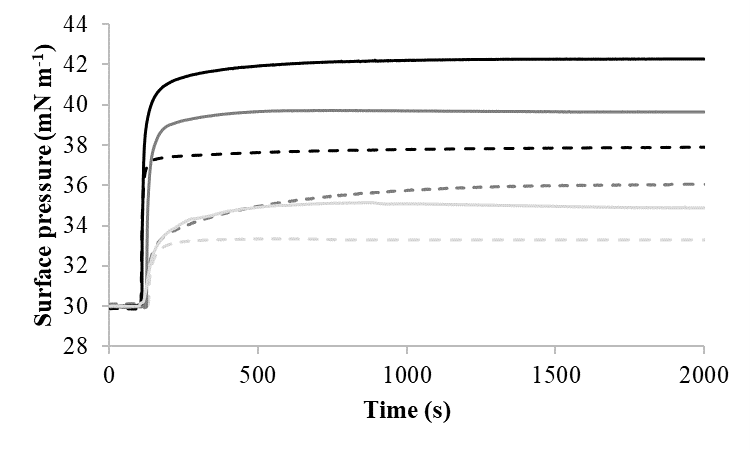

Supplement: Supplementary file 4 — Supplementary file4 Supplementary Figure 4. The interaction of M5-NH2 isoforms with individual CM lipids. Figure 4 shows the change in surface pressure induced by increasing concentrations of M5-NH2 in lipid monolayers formed from solid lines TOCL (black), POPG (dark grey) and POPE (light grey). Also shown are corresponding changes in surface pressure induced by M5-OH with monolayers formed from TOCL (dotted black), POPG (dotted dark grey) and POPE (dotted light grey). In each case, maximal surface pressures were determined (Table 3B).(TIF 53 KB) [file 249_2025_1732_MOESM4_ESM.tif]

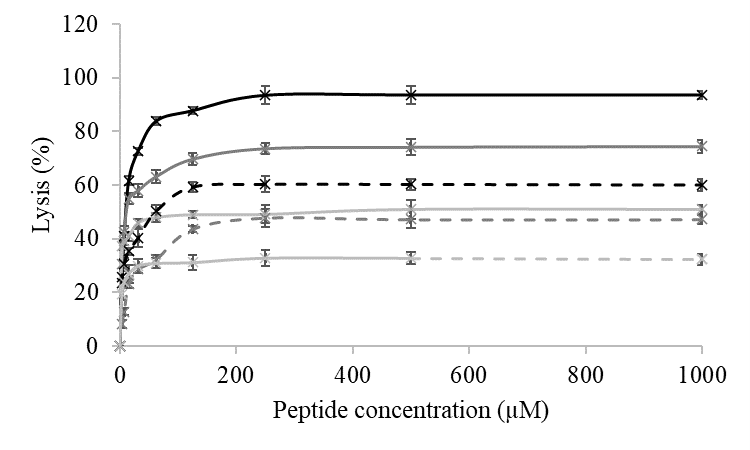

Supplement: Supplementary file 5 — Supplementary file5 Supplementary Figure 5. The membranolytic action of M5-NH2 isoforms against individual CM lipids. Supplementary Figure 5 shows the change in lysis levels induced by increasing concentrations of M5-NH2 with calcein loaded, SUVs formed from TOCL (black), POPG (dark grey) and POPE (light grey). Also shown are corresponding changes in surface pressure induced by M5-OH with calcein loaded SUVs formed from TOCL (dotted black), POPG (dotted dark grey) and POPE dotted light grey). In each case, maximal levels of lysis induced by peptides were determined (Table 3B) and error bars represent the standard deviation. (TIF 57 KB) [file 249_2025_1732_MOESM5_ESM.tif]
